# Identification of a novel human deoxynivalenol metabolite enhancing proliferation of intestinal and urinary bladder cells

**DOI:** 10.1038/srep33854

**Published:** 2016-09-23

**Authors:** Benedikt Warth, Giorgia Del Favero, Gerlinde Wiesenberger, Hannes Puntscher, Lydia Woelflingseder, Philipp Fruhmann, Bojan Sarkanj, Rudolf Krska, Rainer Schuhmacher, Gerhard Adam, Doris Marko

**Affiliations:** 1 University of Vienna, Faculty of Chemistry, Department of Food Chemistry and Toxicology, Währingerstr. 38, 1090 Vienna, Austria; 2 University of Natural Resources and Life Sciences, Vienna (BOKU), Department IFA-Tulln, Konrad-Lorenz-Str. 20, 3430 Tulln, Austria; 3 University of Natural Resources and Life Sciences, Vienna (BOKU), Department of Applied Genetics and Cell Biology, Konrad-Lorenz-Str. 24, 3430 Tulln, Austria; 4 Vienna University of Technology, Institute of Applied Synthetic Chemistry, Getreidemarkt 9, 1060 Vienna, Austria; 5 Josip Juraj Strossmayer University, Department of Applied Chemistry and Ecology, Faculty of Food Technology, 31000 Osijek, Croatia

## Abstract

The mycotoxin deoxynivalenol (DON) is an abundant contaminant of cereal based food and a severe issue for global food safety. We report the discovery of DON-3-sulfate as a novel human metabolite and potential new biomarker of DON exposure. The conjugate was detectable in 70% of urine samples obtained from pregnant women in Croatia. For the measurement of urinary metabolites, a highly sensitive and selective LC-MS/MS method was developed and validated. The method was also used to investigate samples from a duplicate diet survey for studying the toxicokinetics of DON-3-sulfate. To get a preliminary insight into the biological relevance of the newly discovered DON-sulfates, *in vitro*experiments were performed. In contrast to DON, sulfate conjugates lacked potency to suppress protein translation. However, surprisingly we found that DON-sulfates enhanced proliferation of human HT-29 colon carcinoma cells, primary human colon epithelial cells (HCEC-1CT) and, to some extent, also T24 bladder cancer cells. A proliferative stimulus, especially in tumorigenic cells raises concern on the potential impact of DON-sulfates on consumer health. Thus, a further characterization of their toxicological relevance should be of high priority.

The trichothecene deoxynivalenol (DON, vomitoxin) is a frequent contaminant of grains and cereal products world-wide. Since DON constitutes a major issue for food and feed safety, different international expert bodies, including those of the FAO/WHO and EFSA, extensively evaluated its occurrence, exposure, metabolism, and toxicity^1^,^2^,^3^. As a result, regulatory limits were introduced in many countries to manage the concentration of DON in food and feed^4^ and a provisional maximum tolerable daily intake (PMTDI) for DON and its acetylated metabolites of 1 μg/kg body weight was established^1^. Exposure to DON was clearly associated with the consumption of cereals^5^. Recent surveys, applying innovative LC-MS/MS based biomarker approaches, revealed that significant parts of several European populations exceeded the PMTDI in various years^6^,^7^,^8^,^9^,^10^. In humans, DON has been associated with gastroenteritis, whereas in animal models acute DON intoxication causes emesis and chronic low-dose exposure elicits anorexia, growth retardation, immunotoxicity as well as impaired reproduction^11^. Although chronic exposure is evident globally, the effects of low-dose DON exposure on humans are still unknown.

The primary mode of DON action is the efficient inhibition of protein synthesis by binding to eukaryotic ribosomes^12^. Thereby, the synthesis of macromolecules as well as cell signaling, differentiation, and proliferation are impaired. However, DON also activates intracellular protein kinases which mediate selective gene expression and apoptosis^11^. DON has been reported to inhibit several intestinal transporters in the human epithelial intestinal cell line HT-29-D4 while in Caco-2 cells it was found to induce IL-8 secretion^13^. In the human Jurkat T-cell line the induction of oxidative stress was recently confirmed by studying the nuclear translocation of the transcription factor NRF2 and its binding protein KEAP1 as well as by changes in cell levels of reduced glutathione^14^.

It is known since a long time that DON is extensively metabolized to glucuronide conjugates (DON-GlcA) as the predominant products of phase II metabolism in animals^15^. However, the first assay to measure DON and its glucuronides indirectly using enzymatic hydrolysis in human urine was developed by Meky *et al*.^16^ only a decade ago. During the last years, the structures of these conjugates in human urine have been identified with DON-15-GlcA as the major metabolite and minor contributions of DON-3-GlcA and DON-7-GlcA^6^,^7^,^17^. The overall 24 h urinary excretion rate of total DON (i.e. the sum of DON and its glucuronides) was estimated to be on average 72% in a moderately exposed UK population^18^. In the cited study β-glucuronidase from *E. coli* (Type IX-A), which is typically free of sulfatase activity, was employed. This estimate was confirmed in other studies either utilizing direct quantification of glucuronides by LC-MS/MS^19^ or enzymatic hydrolysis and GC-MS instrumentation^20^. Also the bacterial detoxification product deepoxy-DON (DOM-1) was found in lower numbers and concentrations in some studies after enzymatic hydrolysis^21^,^22^ or via a direct approach^10^,^23^. To the best of our knowledge, a DON-sulfate conjugate has not been reported as a human metabolite before. However, literature reports of a tentatively identified DON-sulfate conjugate in sheep urine based on an indirect approach using enzymatic de-conjugation with sulfatase^15^ and samples obtained from chicken tissues^24^ were published. Furthermore, Schwartz-Zimmermann *et al*.^25^ demonstrated DON-3-sulfate as the major DON metabolite in different poultry species and the formation of DOM-3-sulfate. Very recently, DON-3-sulfate and DON-15-sulfate were also unambiguously identified as plant metabolites formed in DON treated wheat^26^ utilizing chemically synthetized reference standards^27^ for structure confirmation and absolute quantitation.

Based on the formation of DON-sulfates as phase II metabolites in animals, we tested the hypothesis that DON may be converted into a sulfate conjugate in humans as well. Hence, we developed a highly sensitive LC-MS/MS method for the direct quantification of DON and its urinary metabolites including DON-sulfates and applied it to two sets of urine samples which have been well-characterized before. We present experimental evidence for the existence of DON-3-sulfate in human urine, which has not been described as a human metabolite of the major trichothecene DON before. Furthermore, we performed a preliminary toxicological characterization of the DON-sulfates which unraveled potential implications on cellular growth.

## Results

### Identification of DON-3-sulfate as novel human metabolite and potential biomarker

**Figure 1:**
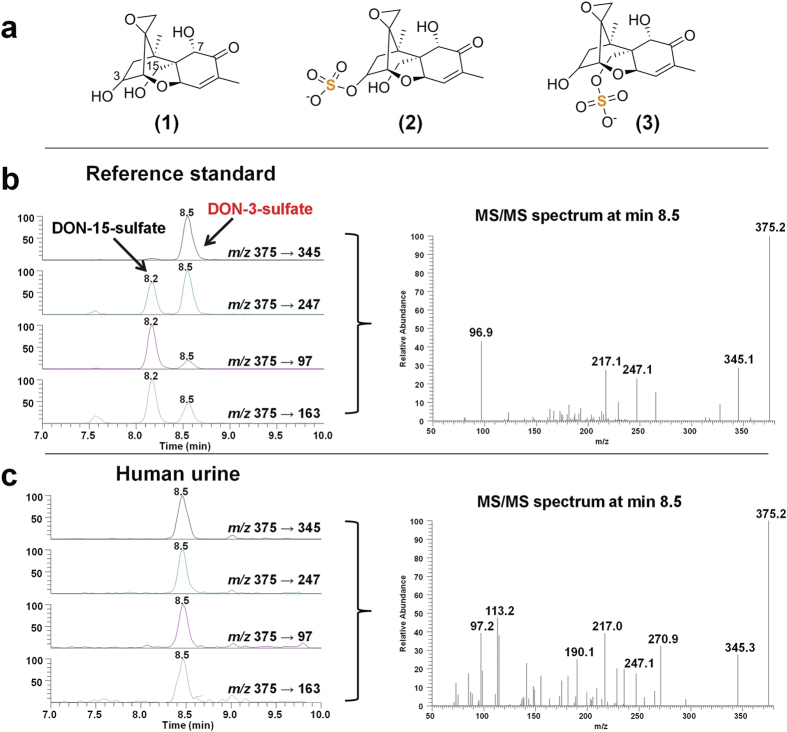
Chemical structures of DON and its sulfates and LC-MS/MS identification of DON-3-sulfate. Structures (**a**) of deoxynivalenol (1), DON-3-sulfate (2) and DON-15-sulfate (3) as well as SRM-chromatograms and MS/MS spectra of authentic reference standards (**b**) and a naturally contaminated urine sample (**c**). The reference (**b**) contains a mixture of DON-3-sulfate and DON-15-sulfate, whereas in the naturally contaminated urine sample (**c**) only DON-3-sulfate is present. Based on a comparison of the retention time and the observed fragments with the standard substance the isomer in the urine sample was identified as DON-3-sulfate. MS/MS scans were recorded at a collision energy of −20 eV.

As illustrated in Fig. 1, DON-3-sulfate was detected in human urine and identified based on comparison with authentic reference standards which have been chemically synthetized and confirmed by NMR before^27^. The retention time as well as the intensity ratio of the selected reaction monitoring (SRM) transitions and the MS/MS spectra identified the detected metabolite as DON-3-sulfate. Whereas glucuronide formation in humans mainly occurs at C-15, sulfates are bound predominantly to the C-3 carbon. Interestingly, no DON-15-sulfate was identified in any of the investigated samples in this study. This means that the unknown human sulfotransferases^28^, mediating conjugation of DON, seem to follow a different stereoselectivity than the involved UDP-glucuronosyltransferases^29^. This is to the best of our knowledge the first report of a DON-sulfate metabolite in any human sample material. Since DON-3-sulfate was only determined in artificially DON-treated wheat but not in any naturally contaminated food sample intended for human consumption and the transfer via chicken meat or eggs^24^,^25^ seems highly unlikely, we propose that the identified conjugate is an endogenous human metabolite produced in the intestine or liver.

### Natural occurrence and excretion rate of DON-3-sulfate in human urine

To investigate the natural occurrence of DON-sulfates, first morning urine samples obtained from Croatian women (n = 40) were analyzed by the newly developed LC-MS/MS based method. DON-3-sulfate was quantified in 28 out of the 40 urine samples (70%). The maximum concentration was 58 μg/L while the average concentration was 4.5 μg/L (0.012 μM), when for samples below the limit of detection (LOD) the half LOD was deployed for average calculation. As mentioned above no DON-15-sulfate was detected in any sample.

Besides the investigation of the natural occurrence of DON-sulfates in human urine, the method was also utilized to re-investigate urine samples from an eight-day duplicate diet survey^19^. This study has been designed initially to unravel the toxicokinetics of DON *in vivo* especially focusing on the formation of glucuronide conjugates. The DON-3-sulfate metabolite was determined in this set of samples frequently as well and its urinary 24 h excretion rate was estimated to be approximately 4% of the DON quantity ingested through the contaminated food (Table 1). The fast elimination of the sulfate conjugate was verified by its absence in the urine sample obtained on day seven, the first day after the consumption of DON contaminated food was stopped.

### LC-MS/MS method development and validation

The MS/MS parameters of DON-sulfates as well as the other analytes (Table 2) included in the method were optimized in both, the positive and the negative ESI mode. All analytes investigated in this study yielded higher absolute signals and better signal to noise ratios in the negative ionization mode. To differentiate between the two isomers the fragment ion at *m/z* 345 (−30 amu) was used. This corresponds to [M-CH_2_O-H]^−^ with a loss of CH_2_O from the -CH_2_OH group attached to the carbon at the C-6 position of the DON-3-sulfate as described before^26^.

The eluents were optimized in order to maximize the retention, recovery and signal to noise ratio of all analytes, however, DON-sulfates were regarded as the most relevant targets. One important objective was to chromatographically baseline separate the DON-sulfate and DON-glucuronide isomers. This task was successfully accomplished by careful optimization of the mobile and stationary phases. Acidified methanolic eluents and the same stationary phase with biphenyl chemistry have been reported recently to exhibit excellent separation of DON and its polar conjugates^25^. Since higher concentrations of acetic acid resulted in decreased signal intensities only a low concentration (0.05%) was chosen for the final method.

The proposed method was validated thoroughly to estimate the linear range, matrix effects, intra- and interday precision, selectivity, as well as the LOD and limit of quantification (LOQ) values. Detailed results are presented in Supplementary Table 1. The method proved to be linear over three orders of magnitude when measuring reference standards in pure solvent. It has been reported before that DON and its polar conjugates are prone to severe matrix effects in biological samples^23^,^30^,^31^. Interestingly, DON-sulfates have been described being susceptible to signal enhancement rather than ion suppression during electrospray ionization in samples derived from animal material^25^ and wheat samples^26^. This behavior was confirmed in human urine in this work albeit in a less pronounced manner with acceptable and very stable apparent recoveries ranging from 107–111% and 114–117% for DON-3-sulfate and DON-15-sulfate, respectively. Also the intra- and interday precision with relative standard deviations of 6–15% and 5–12%, respectively can be regarded as acceptable when taking the fast and effective sample preparation and the challenging biological matrix into account. The obtained LODs (DON-3-sulfate: 0.45 μg/L; DON-15-sulfate: 0.35 μg/L; see Supplementary Table 1) were judged to be applicable to quantify even low DON exposures. The retention times were stable with a maximum shift of less than 1.2% for DON-sulfates which is typically regarded as acceptable for LC separations. Overall, the results clearly indicated that the chosen ‘dilute and shoot’ approach was feasible and did not require any further sample clean-up or enrichment step.

### Effect of DON and its sulfates on the translation efficiency in mammalian cells

**Figure 2:**
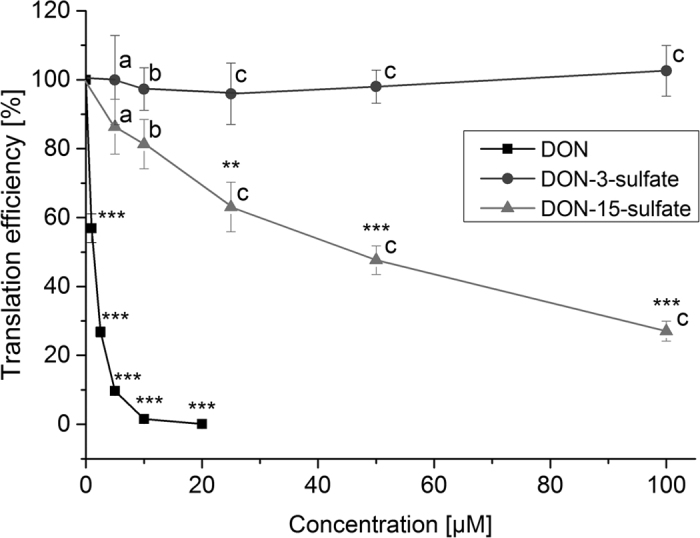
Effects of DON, DON-3-sulfate and DON-15-sulfate on translation by mammalian ribosomes. All data were tested on normality by the Shapiro Wilk test. Effects of different concentrations of DON and DON-sulfates were tested on significant differences to the water control by One-Way ANOVA and are indicated by ***(p < 0.001) and **(p < 0.01). Significant differences of effects between DON-sulfates and 5 μM (**a**), 10 μM (**b**) and 20 μM (**c**) DON (p < 0.001) were tested by Student’s *t*-test. Results represent the mean ± SE of six independent experiments.

Since the primary mode of DON and trichothecene action is the inhibition of protein biosynthesis by eukaryotic ribosomes, we tested whether a rabbit reticulocyte based *in vitro* translation assay was affected by either sulfate conjugate (Fig. 2). While 1.5 μM DON reduced production of the reporter protein to 50% and translation was completely inhibited in the presence of 20 μM DON, DON-3-sulfate did not inhibit *in vitro* translation at concentrations of up to 100 μM. DON-15-sulfate was shown to be a moderate inhibitor of mammalian ribosomes with an IC_50_ of about 47 μM.

### Effect of DON and its sulfates on cell growth (sulforhodamine B assay)

**Figure 3:**
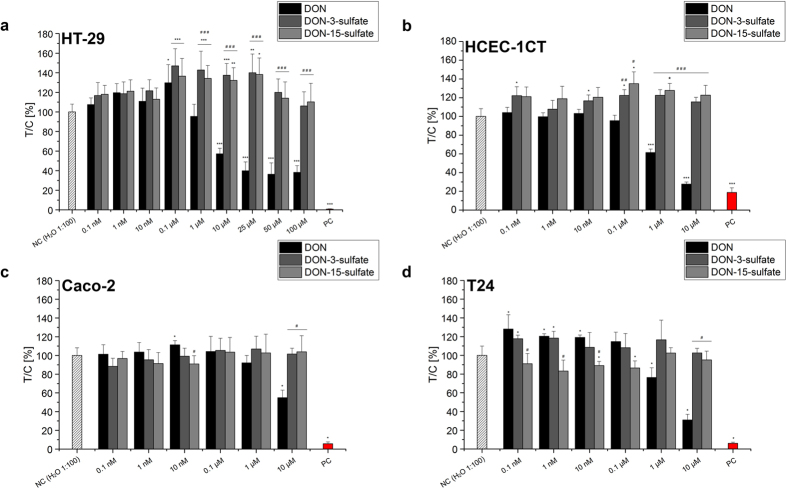
Effects of DON (black bars), DON-3-sulfate (dark grey bars) and DON-15-sulfate (light grey bars) on HT-29 (**a**), HCEC-1CT (**b**), Caco-2 (**c**) and T24 (**d**) cells in the sulforhodamine B (SRB assay). *Indicates significant differences compared to negative control (NC (H_2_O 1:100)); *p < 0.05; **p < 0.01; ***p < 0.001). ^#^Indicates significant differences in comparison to the values of DON at the same concentration (^#^p < 0.05; ^##^p < 0.01; ^###^p < 0.001). Values are expressed as mean of at least 3 independent experiments performed in quadruplicate ± SE PC: positive control.

Incubation of intestinal (Fig. 3a,b,c) and bladder cells (Fig. 3d) with DON *in vitro* resulted in a concentration dependent cytotoxicity. A significant decrease of cell viability was detectable starting from the concentration of 1 μM for HCEC-1CT and T24 cells (Fig. 3b,d) and starting from 10 μM in HT-29 and Caco-2 cells (Fig. 3a,c). In addition, in a limited and low concentration range, DON triggered the proliferation of the tumor cells tested in the present study (HT-29: 0.1 μM; Caco-2: 10 nM; T24: 0.1–10 nM) but not in the non-transformed human colonic epithelial cells HCEC-1CT. In line with the data of the translation inhibition assay, DON-3-sulfate did not exert cytotoxic effects in any of the test systems, while DON-15-sulfate induced a slight decrease of cell viability in T24 cells when incubated at low concentrations of 10 nM and 0.1 μM.

Interestingly, the two sulfate conjugates demonstrated a marked proliferative stimulus on HT-29 colon carcinoma cells in a concentration range between 0.1 and 25 μM (Fig. 3a). This effect was confirmed in HCEC-1CT and T24 cells albeit less pronounced (Fig. 3b,d) while it was not significant in Caco-2 cells (Fig. 3c). For the primary human colon epithelial cells HCEC-1CT the increase was present at concentrations of 0.1 and 10 nM as well as 0.1 μM in cells incubated with DON-3-sulfate. For DON-15-sulfate the effect was found at concentrations of 0.1 and 1 μM. In agreement with the data obtained in intestinal HT-29 and HCEC-1CT cells, DON-3-sulfate triggered a proliferative stimulus also in urinary bladder T24 cells at concentrations of 0.1 and 1 nM.

### Cellular metabolism

To evaluate if potential effects of DON-sulfates may arise from hydrolysis to the parent compound under the chosen *in vitro* conditions, the cellular metabolism of the compounds was preliminarily studied in the intestinal cell line showing the most potent effect. Since free DON was neither detected in the supernatant nor in the cell lysate of HT-29 cells incubated with 10 μM of DON-3-sulfate or DON-15-sulfate, we concluded that all effects observed in the applied *in vitro* toxicity assays are caused predominantly by the conjugate itself. Hydrolysis of sulfates did not occur and the sulfates seemed to be stable compounds in general.

## Discussion

This is to the best of our knowledge the first report of a DON-sulfate metabolite in any human sample. Based on the chromatographic retention behavior and the MS/MS spectra displayed in Fig. 1, the isomer occurring in human urine was identified as DON-3-sulfate. In principle, also the formation of DON-7-sulfate might be possible. However, the unreactivity of the C7 position to chemical sulfation has been demonstrated before^27^ and it is unlikely that a potentially occurring DON-7-sulfate, for which no reference standard is available yet, co-elutes with DON-3-sulfate under the tailored chromatographic conditions and shows the same MS/MS spectrum.

DON-3-sulfate was found to be present in 70% of the investigated samples obtained from Croatian women with a high maximum concentration of 58 μg/L, corresponding to 0.15 μM. In addition, it was detected frequently in a set of samples from an *in vivo* toxicokinetics study utilizing urine samples obtained from a male Austrian volunteer. Thereby, the urinary 24 h excretion rate was estimated to be approximately 4% of the DON quantity ingested through the consumption of contaminated food (Table 2). This likely indicates that sulfation is a minor metabolic pathway compared to glucuronidation^19^,^32^, although the fraction of DON-3-sulfate excreted in the bile was not estimated. However, the contribution in human urine is higher than the 2% reported for sheep^15^. Besides, it might be possible that sulfates but not glucuronides are transferred through the cell membrane by specific transporters. In the investigated population sulfation was more relevant than de-epoxidation as no DOM-1 was detected in any sample. DOM-1 was first demonstrated in the urine of French farmers, representing on average <5% of the total urinary DON in individuals with detectable DOM-1 levels^21^. Since then it was demonstrated in a limited number of studies mainly in its glucuronide form^10^,^22^,^23^.

The sulfotransferases responsible for mammalian xenobiotic metabolism are cytosolic enzymes forming a gene superfamily. Differences in substrate specificity between the different sulfotransferases can be relevant for tissue-specific toxicological effects^28^. Ten distinct human sulfotransferase forms are known, however, currently it is unknown which gene product is mediating the conjugation with DON. This information would also be of relevance since the distribution of sulfotransferases may strongly differ between tissues. As one example hP-PST (human phenol sulfotransferases) exhibit high expression levels in the liver while it is detected typically in lower levels in other tissues.

Meky *et al*.^16^ reported that rat urine incubated with sulfatase resulted in no change of DON related chromatographic peaks. Hence, in the past most bio-monitoring studies focusing on the indirect quantification of DON employed β-glucuronidase from *E. coli*, which is essentially free of sulfatase activity^7^,^18^,^33^. Based on the identification of DON-3-sulfate in this study the use of β-glucuronidase/sulfatase from from *Helix pomatia* is recommended for future studies as already described by some groups^34^,^35^,^36^.

While DON-3-sulfate does not inhibit *in vitro* protein synthesis at concentrations up to 100 μM (Fig. 2) DON-15-sulfate was found to be a moderate inhibitor of mammalian ribosomes with an IC_50_ of about 47 μM. The inhibition observed in this experiment is slightly lower than that observed on wheat ribosomes, where the IC_50_ of DON-15-sulfate was about 66 μM^26^. When compared to DON, these figures demonstrate that DON-sulfates can be regarded as detoxification products with respect to their effect on protein translation.

In order to enable a preliminary characterization of the biological activity of the DON-sulfates in comparison to the parent compound DON, additional cytotoxicity experiments were performed. Four cell lines derived from the intestinal tract (HT-29, HCEC-1CT, Caco-2) and from the urinary bladder (T24) were selected to give a comprehensive overview. In agreement with the effect of the three compounds on mammalian ribosomes (Fig. 2), DON was cytotoxic in all the tested cell types while DON-3-sulfate did not exert any toxic effect and DON-15-sulfate showed only a limited effect in T24 cells. Intriguingly, when incubated in the nanomolar range DON triggered a proliferative stimulus in the cells of cancerous origin used in the present study.

In addition, the two sulfate metabolites demonstrated a distinct proliferative stimulus on human colorectal adenocarcinoma HT-29 cells over a concentration range between 0.1 and 25 μM. This effect was present, even if more limited, also in the non-transformed HCEC-1CT and, for the DON-3-sulfate, also in the urinary bladder carcinoma cells T24. The effect was present at very low concentrations, starting from 0.1 μM in HT-29 cells and even lower for the T24 cells (from 0.1 nM in the cells incubated with DON and DON-3-sulfate) and HCEC-1CT (from 0.1 nM in the cells incubated with DON-3-sulfate). This is of particular interest since this concentration range seems to be coherent with the concentration of the urinary DON metabolites that can be found also in the bladder *in vivo* as suggested by the urinary concentrations reported in this paper. Taking into account that several recent bio-monitoring studies reported on individuals exceeding the proposed PMTDI established for DON^6^,^7^,^8^,^9^,^10^,^37^ and the frequent occurrence of DON-3-sulfate in the urine of exposed individuals in the study at hand, this highlights the urgent need for further studies and a deeper toxicological characterization of DON-sulfates. It should also be considered that we were able to demonstrate the capacity of wheat plants to form both, DON-3-sulfate and DON-15-sulfate conjugates in a previous study^26^. In a wheat suspension culture additionally 15-acetyl-DON-3-sulfate was reported very recently^38^. Hence, it seems plausible that this new class of masked/modified mycotoxins might enter the body via contaminated food in addition to the proposed endogenous production of DON-3-sulfate in the human body.

A proliferative effect of DON on tumor cells at very low concentrations has been reported for the parent compound DON in recent experiments as well^39^,^40^. However, according to literature and confirmed by our data this effect disappears once the cytotoxicity of DON outstrips the growth stimulus at a concentration of 1 μM. The sulforhodamine B (SRB) assay applied in this work measures the cellular protein content and is a standard assay of the National Cancer Institute for *in vitro* anticancer-drug screening. It provides a sensitive measure of cytotoxicity induced by drugs or xenobiotics and is frequently used to quantify clonogenicity^41^.

DON has been considered to be non-carcinogenic (Group 3) by the International Agency for Research on Cancer for a long time^42^. Even though the impact of DON and its metabolites on the growth of different cells types remains to be clarified with respect to the mechanisms sustaining it and future risk assessment, this is the first report on DON metabolites which potentially promote the cellular growth at concentrations occurring *in vivo* due to widespread chronic exposure.

In summary, we demonstrated for the first time that DON-3-sulfate is a human metabolite of the abundant food contaminant DON. Using a newly developed, highly sensitive and selective LC-MS/MS method this new potential biomarker was quantified in the majority of tested urine samples. To evaluate the potential consequences of this unexpected finding for consumers of mycotoxin contaminated food, preliminary toxicological testing was performed. Interestingly, and maybe of high importance for public health and future DON risk assessment, it was found that the DON-sulfates can trigger cellular proliferation *in vitro* in a concentrations range that seems to be relevant *in vivo* as suggested by the obtained urinary concentrations of DON-3-sulfate.

## Methods

### Chemicals and reagents

Methanol, acetonitrile, acetic acid and water were all purchased from Sigma (Fluka; Vienna, Austria) and of LC-MS grade. DON-3-sulfate and DON-15-sulfate were synthesized using a sulfuryl imidazolium salt as described by Fruhmann *et al*.^27^ whereas DON-3-glucuronide (DON-3-GlcA) was synthesized by an optimised Königs-Knorr procedure using acetobromo-α-D-glucuronic acid methyl ester as glucuronyl-donor^43^. DON and DOM-1 were purchased from Romer Labs Diagnostic GmbH (Tulln, Austria). Solid substances were dissolved in water for *in vitro* experiments and in pure methanol (DON-3-sulfate, DON-15-sulfate, DON-3-GlcA) or acetonitrile (DON, DOM-1) for analytical purpose and stored at −20 °C. A combined multi standard working solution for preparation of calibrants and spiking experiments was prepared in acetonitrile containing 2 mg/L of DON-3-sulfate, DON-15-sulfate, DON, and DOM-1 as well as 4 mg/L DON-3-GlcA.

### Urine samples

The samples used in this study originated from two different experiments. To generally investigate the occurrence of DON-sulfates in a population exposed to high levels of DON, first morning urine samples obtained from Croatian women (n = 40) were utilized. Volunteers were all healthy, non-smoking pregnant women in their final trimester of gestation who resided in the eastern area of Croatia (from and around the city of Osijek; age: 26–33 years old). These samples have previously been tested on multiple mycotoxin biomarkers using an advanced LC-MS/MS method^6^ as well as on ochratoxin A and ochratoxin alpha using HPLC-FLD^44^. They partly exhibited high concentrations of DON (max. 275 μg/L), DON-3-GlcA (max. 298 μg/L), and DON-15-GlcA (max. 1238 μg/L) and are thus ideally suited to screen for novel metabolic products of DON. Samples were taken in February 2011 and stored at −20 °C until analysis. Informed consent was obtained from all participants. The study was approved by the Ethics Committee of the Faculty of Food Technology, University Josip Juraj Strossmayer Osijek and the measurements were carried out in accordance with the approved guidelines.

The second set of samples originated from an *in vivo* case study which investigated human DON and zearalenone (ZEN) metabolism in detail through the analysis of urine samples obtained from one Austrian volunteer following a naturally contaminated diet containing 138 μg DON and 10 μg ZEN over a period of four days^19^. Sulfate conjugates were not included in the original study due to a lack of an authentic reference standard at that time. The study was conducted on a 27 year old, healthy male volunteer whose diet consisted of cereals with wheat bran for breakfast, maize porridge (including maize flour) for lunch and bread, beer and pop-corn in the evening as described in detail before^19^. For calculating average DON-sulfate excretion rates 24 h urine samples were used in the study at hand. Samples were taken in July 2011 and stored at −20 °C until analysis. This study was approved by the ethics commission of the government of Lower Austria. Measurements were carried out in accordance with the approved guidelines after informed consent was obtained.

### Sample preparation

The time- and cost-effective sample preparation procedure chosen was based on a protocol for the simultaneous quantification of multiple mycotoxins and metabolites^45^. In brief, samples were allowed to reach room temperature, centrifuged for 3 min at 10.000 rpm, and diluted 1:10 with a neat dilution solvent (ACN/H_2_O: 10/90).

### LC-MS/MS instrumentation

Method development, validation, and sample analysis was carried out using a Thermo TSQ Vantage LC-MS/MS triple quadrupole system (Thermo, San Jose, CA, USA) coupled to an Accela 1250 LC system. Data acquisition was performed using the Xcalibur software (version 3.0) whereas the evaluation of data was done using LCquan (version 2.9). The mass spectrometer was equipped with a heated electrospray (hESI) interface which was operated in negative ionization mode. Nitrogen was used as drying and argon as collision gas. The parameters of the ion source are reported in Supplementary Table 2.

Analytes were separated on a Kinetex Biphenyl column (3.0 × 150 mm, Phenomenex, Torrance, CA, US) with 2.6 μm particle size and a SecurityGuard ULTRA pre-column (Phenomenex). Gradient elution at 40 °C was performed within 17 min. Eluent A (H_2_O/MeOH; 9/1) and eluent B (MeOH) both contained 0.05% acetic acid and the flow rate was set to 400 μL/min. After an initial time period of 1.0 min at 100% A, the percentage of B was linearly raised to 16% until minute 10.0. Then, eluent B was raised to 95% until minute 12.0 followed by a hold-time of 2.0 min and subsequent 3.0 min column re-equilibration at 100% A. A volume of 10 μL of the diluted samples corresponding to 1 μL undiluted urine was injected. ESI-MS/MS was performed in selected reaction monitoring (SRM) mode for all analytes investigated in this study. At least two individual transitions were monitored for each analyte. Analyte dependent MS/MS parameters were optimized via direct infusion of reference standards. Quantification of all analytes for which reference standards were available was done by external calibration curves (1/x weighted) as described in the validation section below and all results were corrected for the apparent recovery of the respective analyte.

Two QC samples were included in each batch of 20 samples within an LC-MS/MS measurement sequence. One was the same pooled blank urine used during validation while the other was the blank urine spiked with working standard solution. The results of the spiked QC sample required to be within 15% of the assigned values. In case of non-accordance the whole sequence was rejected for the affected analyte.

### Method validation

In-house validation of the developed method was carried out to determine the parameters linear range, precision, recovery, selectivity, and sensitivity. Intra- and interday precision as well as the apparent recovery of analytes were evaluated by measurements of a pooled blank urine sample spiked with the working solution at three concentration levels: Low (3 μg/L), middle (30 μg/L) and high (300 μg/L). When taking the urine dilution into account this corresponds to 0.3, 3, and 30 μg/L, covering a wide range of concentrations. Spiking experiments were performed in triplicate and on three different days. Intra- (n = 9) and interday (n = 27) precision were expressed as the relative standard deviation of the obtained recoveries for each metabolite. The selectivity of the chosen product ions was evaluated throughout method development and validation and was continued during the application of the method to experimental samples. LOD and LOQ values were calculated from chromatograms of spiked blank urine samples based on a signal to noise ratio of 3:1 and 6:1, respectively. Calibration curves (1/x weighted) were constructed from peak areas of the reference standards in solvent plotted against their concentrations. Each calibration was carried out at seven concentration levels covering three orders of magnitude. The calibration range was 0.1–100 μg/L for all analytes.

### Effect of DON and its sulfates on the translation efficiency of mammalian cells

The TnT^®^ T7 Coupled Reticulocyte Lysate System (Promega, Madison WI, USA) was used for *in vitro* translation experiments. The assays were performed as described before^46^ with minor modifications. Six independent experiments were performed. All data were tested on normality by the Shapiro Wilk test. Different doses of DON and DON-sulfates were tested on significant differences to the water control by One-Way ANOVA. Significant differences between selected DON and DON-sulfate concentrations were tested by Student’s *t*-test.

### Cell culture

HT-29, Caco-2 (C2BBe1 clone) and T24 (ATCC^®^ HTB4™) cells were purchased from ATCC. HT29 and Caco-2 cells were cultivated in DMEM supplemented with 10% fetal calf serum (FCS) and 1% penicillin/streptomycin (50 U/mL). T24 cells were cultivated in McCoy’s 5A Medium (1X) containing 10% FCS. HCEC-1CT cells^47^ were kindly provided by Prof. Jerry W. Shay (UT Southwestern Medical Center, Dallas, TX, USA) and cultivated in a basal medium obtained from DMEM high glucose mixed with 10X medium 199 (2%) and supplemented with cosmic calf serum (2%), hepes 20 mM, gentamicin (50 μg/ml), insulin-transferrin-selenium-G supplement (10 μl/ml), recombinant human EGF (20 ng/ml), and hydrocortisone (1 μg/ml)^48^. Cell culture media and supplements were purchased from GIBCO Invitrogen (Karlsruhe, Germany), Lonza Group Ltd (Basel, Switzerland), Sigma-Aldrich Chemie GmbH (Munich, Germany) and Sarstedt AG & Co (Nuembrecht, Germany), VWR International GmbH (Vienna, Austria), Fisher Scientific (Austria) GmbH (Vienna, Austria), Szabo-Scandic HandelsgmbH & Co KG (Vienna, Austria). For cell cultivation and incubations humidified incubators at 37 °C and 5% CO_2_ were used and cells were routinely tested for absence of mycoplasma contamination.

### Effect of DON and its sulfates on cell viability (sulforhodamine B assay)

In order to provide an initial characterization of the biological effects of the DON-sulfates at cellular level SRB experiments were performed as described previously^41^,^49^. HT-29, HCEC-1CT, Caco-2 and T24 cells were seeded in 96-wells plates and incubated with different concentrations of the parent compound or the metabolites for 24 h. At the end of the incubation cells were rinsed twice with PBS (100 μL) and fixed for 30 min at 4 °C with 50 μL of 50% trichloroacetic acid (TCA) per well. To remove TCA cells were repetitively rinsed with water and 100 μL of SRB reagent (SRB 0.4% in 1% acetic acid) were added to each well. After 1 h of incubation, stained cells were rinsed with acetic acid (1%) and water to remove the unbound SRB and, subsequently, the protein-bound SRB was solubilized with 100 μL Tris (10 mM). Single wavelength absorbance (570 nm) was read on a Cytation 3 Imaging Multi Mode Reader (BioTek, Bad Friedrichshall, Germany). Results are presented as mean of at least three independent experiments performed in quadruplicate ± SE and analyzed applying the Kruskal-Wallis-ANOVA test with OriginPro software (version 9.1).

### Cellular metabolism

HT-29 cells were seeded in 24-well plates (50.000 cells per well). After 48 h cells were incubated in triplicate with 10 μM of either DON, DON-3-sulfate, or DON-15-sulfate for 0, 3, 24, and 48 h. After the respective time the supernatant and the cell lysate were analyzed separately to evaluate if deconjugation occurred under cell culture conditions. The supernatant was diluted 1:1 (v/v) with MeOH, centrifuged (18.000 rpm, 5 min) and 10 μL of the diluted supernatant were injected into the LC-MS/MS system. The HT-29 cells were washed with ice cold PBS and detached from the culture plates with 100 μL trypsin. After addition of 100 μL ice cold MeOH the cells in suspension were disrupted by shock freezing in liquid nitrogen twice. The cell lysate was centrifuged (18.000 rpm, 10 min) and 100 μL of the supernatant were transferred to an autosampler vial with micro insert and subsequently analyzed by LC-MS/MS.

### Ethics statement

All experiments involving human urine samples were approved by the responsible ethics commission. The study involving Croatian samples was approved by the Ethics Committee of the Faculty of Food Technology, University Josip Juraj Strossmayer Osijek whereas the Austrian study was permitted by the ethics commission of the government of Lower Austria.

## Additional Information

**How to cite this article**: Warth, B. *et al*. Identification of a novel human deoxynivalenol metabolite enhancing proliferation of intestinal and urinary bladder cells. *Sci. Rep.* **6**, 33854; doi: 10.1038/srep33854 (2016).

## Supplementary Material

Supplementary Information

## Figures and Tables

**Table 1:** *In vivo* metabolism of DON to DON-3-sulfate in an eight-day duplicate diet case study^19^.

	DON intake^a^ in μg/d and (μmol/d)	Urine excretion [L]	DON-3-sulfate^b^ [μg/L]	DON-3-sulfate^b^ in μg/d and (μmol/d)	D3S excretion rate [%]^c^
Day 1	—	2.2	n.d.	n.d.	n.d.
Day 2	—	1.8	n.d.	n.d.	n.d.
Day 3	138 (0.47)	2.2	2.8	6.0 (0.02)	4.3
Day 4	138 (0.47)	2.7	1.0	2.8 (0.01)	2.1
Day 5	138 (0.47)	2.3	2.2	4.8 (0.02)	3.5
Day 6	138 (0.47)	2.5	2.3	5.9 (0.02)	4.3
Day 7	—	2.4	n.d.	n.d.	n.d.
Day 8	—	1.6	n.d.	n.d.	n.d.
Average	138 (0.47)	2.4	2.1	4.9 (0.02)	3.5

A ‘high DON diet’ predominantly consisting of contaminated cereals was consumed during days 3–6 while days 1–2 and 7–8 were clearing periods.

^a^Daily DON intake without taking masked forms (3-acetyl DON, 15-acetyl-DON, DON-3-glucoside) into account.

^b^Expressed as DON equivalents.

^c^Excretion rate was calculated as follows: Excreted quantity DON-3-sulfate in μmol/DON intake in μmol * 100.

**Table 2:** Optimized ESI-MS and ESI-MS/MS parameters as obtained during method optimization.

Analyte	RT [min]	Precursor ion [*m/z*]	Ion species	Product ions^a^ [*m/z*]	Relative intensity^b^	CE^a^^,^^c^ [eV]	S-lens
DON	9.4	355.1	[M + Ac]^−^	265.2/247.2	29%	−17/−19	75
DON-3-sulfate	8.5	375.0	[M−H]^−^	345.0/247.0	59%	−21/−24	100
DON-15-sulfate	8.2	375.0	[M−H]^−^	97.0/163.1	22%	−35/−40	100
DON-3-glucuronide	8.8	471.1	[M−H]^−^	265.0/175.0/441.0	93%/37%	−27/−30/−23	150
DON-15-glucuronide	9.0	471.1	[M−H]^−^	265.0/175.0/441.0	27%/3%	−27/−30/−23	150
Deepoxy-DON	12.6	339.1	[M + Ac]^−^	249.0/59.0	106%	−15/−23	62

^a^Values are given in the order quantifier ion/qualifier ion/qualifier ion 2 (in case of glucuronides).

^b^Signal intensity of the qualifier transition in relation to the quantifier (qualifier/quantifier × 100).

^c^Collision energy.
