# Supplementary material for: Identification of a novel human deoxynivalenol metabolite enhancing proliferation of intestinal and urinary bladder cells
Source: Sci Rep. 2016 Sep 23;6:33854. doi: 10.1038/srep33854 (PMC5034337; doi:10.1038/srep33854)
Supplement: Supplementary Information [file srep33854-s1.pdf]

Identification of a novel human deoxynivalenol metabolite enhancing proliferation of intestinal and urinary bladder cells

Benedikt Warth[1,2,†,*], Giorgia Del Favero[1], Gerlinde Wiesenberger[3], Hannes Puntscher[1], Lydia Woelflingseder[1], Philipp Fruhmann[3,4], Bojan Sarkanj[2,5], Rudolf Krska[2], Rainer Schuhmacher[2], Gerhard Adam[3], Doris Marko[1]

[1]University of Vienna, Faculty of Chemistry, Department of Food Chemistry and Toxicology, Währingerstr. 38, 1090 Vienna, Austria

[2]University of Natural Resources and Life Sciences, Vienna (BOKU), Department IFA-Tulln, Konrad-Lorenz-Str. 20, 3430 Tulln, Austria

[3]University of Natural Resources and Life Sciences, Vienna (BOKU), Department of Applied Genetics and Cell Biology, Konrad-Lorenz-Str. 24, 3430 Tulln, Austria

[4]Vienna University of Technology, Institute of Applied Synthetic Chemistry, Getreidemarkt 9, 1060 Vienna, Austria

[5]Josip Juraj Strossmayer University, Department of Applied Chemistry and Ecology, Faculty of Food Technology, 31000 Osijek, Croatia

[†]Present address: The Scripps Research Institute, Center for Metabolomics and Mass Spectrometry, 10550 North Torrey Pines Road, La Jolla, California 92037, USA

*Dr. Benedikt Warth, Department of Food Chemistry and Toxicology, University of Vienna, Währingerstr. 38, 1090 Vienna, Austria

Email: benedikt.warth@univie.ac.at

Tel: +43-1-4277-70805

Supplementary information

Supplementary Table 1. Performance parameters as obtained during in-house validation

Analyte	Recovery[a] [%]			Precision intra- (n=9)/interday (n=27) RSD [%][c]		LOD[d] [µg/L]	LOQ[e] [µg/L]
	Low[b] (3 µg/L)	Middle[b] (30 µg/L)	High[b] (300 µg/L)				
**Deoxynivalenol**	102	95	91	10	8	0.9	1.8
**DON-3-sulfate**	111	107	110	8	7	0.45	0.9
**DON-15-sulfate**	117	114	115	6	5	0.35	0.7
**DON-3-glucuronide**	106	99	102	12	11	1.8	3.6
**Deepoxy-DON**	96	78	82	15	12	1.2	2.4

[a]Apparent recovery; determined from the mean of spiked samples at three different levels (n=9).

[b]For DON-3-glucuronide the spiking concentrations was a factor two higher

[c]Mean relative standard deviation (RSD) from each three determinations at three concentration levels on one day (intraday) or three days (interday).

[d]LOD in matrix taking the urine dilution into account based on a S/N ratio of 3:1

[e]LOQ in matrix taking the urine dilution into account based on a S/N ratio of 6:1

Supplementary Table 2. Ion source settings on the TSQ Vantage triple quadrupole instrument operated in the negative ionization mode

Settings	TSQ Vantage
**Source temperature (°C)**	400
**Capillary voltage (kV)**	−3.1
**Collision gas pressure (mTorr)**	1.4
**Sheath gas pressure (Arb)**	60
**Ion sweep gas pressure (Arb)**	0
**Aux gas pressure (Arb)**	20
**Capillary temperature (°C)**	300
